# Older Perpetrators of Domestic Violence: Mixed-Effects Logistic Regression Analysis of Police Records

**DOI:** 10.2196/75993

**Published:** 2025-09-29

**Authors:** Sharon Reutens, Emaediong Akpanekpo, George Karystianis, Adrienne Withall, Tony Butler

**Affiliations:** 1 Faculty of Medicine UNSW Sydney Sydney Australia; 2 UNSW Sydney Sydney Australia

**Keywords:** dementia, domestic violence, abuse of older people, abuse, police, text mining, older

## Abstract

**Background:**

Domestic violence (DV) among older adults is an understudied area, often overlapping with abuse of older people, intimate partner violence, and behavioral and psychological symptoms of dementia.

**Objective:**

This study aimed to examine the characteristics of older persons of interest—individuals suspected or charged with a DV offence—and survivors involved in police-attended DV events in New South Wales, Australia, and assess associations with physical and nonphysical abuse.

**Methods:**

Police records of 10,708 DV events involving 8247 adults aged ≥55 years who were identified as persons of interest from 2005 to 2016 were analyzed using text mining. A 3-level mixed-effects logistic regression model was used to identify predictors of physical and nonphysical abuse.

**Results:**

Physical abuse formed a greater proportion of all abuse committed by female persons of interest aged >65 years compared to female persons of interest aged between 55 and 64 years and male persons of interest; however, after stratified analysis, female persons of interest had similarly elevated odds of physical abuse perpetration to male persons of interest. Other factors associated with increased odds of perpetrating physical abuse were persons of interest with dementia and alcohol-related events. Dementia increased the odds of combined physical and nonphysical abuse. Substance use disorders increased the odds of events with combined physical and nonphysical abuse.

**Conclusions:**

The findings of this study suggest that DV, including physical violence, is an important issue in later life. Alcohol is a situational factor, and dementia is associated with perpetration and exposure to violence. The study highlights the need for clinicians to evaluate the risk of violence and exposure to violence in patients with dementia and for policy interventions targeting alcohol and substance use in older adults.

## Introduction

Domestic violence (DV) is a pervasive problem experienced by individuals across different ages, sexes, cultural backgrounds, and socioeconomic status. In Australia, the New South Wales Crimes (Domestic and Personal Violence) Act 2007 defines DV as any physical, sexual, financial, emotional or psychological abuse perpetrated within a current or past intimate partner and family relationship and includes DV against household residents and kin. DV is estimated to affect 20% of Australian people aged 18 years or older [1]. Survivors experience a wide range of adverse physical and mental health outcomes (eg, posttraumatic stress disorder, suicidal behavior, substance abuse, and injuries) or death as well as socioeconomic impacts from controlling behaviors about finances and the risk of homelessness [2-4].

There is a paucity of literature on DV in older survivors and perpetrators that is further complicated because violence in this age group can fall into several categories such as abuse of older people, intimate partner violence (IPV), or, if a person has dementia, perceived as behavioral and psychological symptoms of dementia (BPSD). The World Health Organization defines IPV as “any behaviour within an intimate relationship that causes physical, psychological or sexual harm to those in the relationship” [5], while abuse of older people is seen as “a single or repeated act or lack of appropriate action, occurring within any relationship where there is an expectation of trust, which causes harm or distress to an older person” [4]. BPSD is “an umbrella term for a heterogenous group of non-cognitive symptoms that are almost ubiquitous in dementia” [6]. While the categories are useful for examining factors related to the relationship between perpetrator and survivor (abuse of older people and IPV) and problem behaviors in people with dementia (BPSD), these divisions have hampered the understanding of DV committed by older people.

Studies of DV with a focus on older people suggest that while physical abuse declines with age, emotional abuse does not [7-9]. However, if research excludes perpetrators and survivors living with dementia, the overall picture of violence perpetrated by older people will be incomplete. For instance, it is known that people with dementia are more likely to be abused if their care needs are greater [10,11], and aggression by people with dementia is common, with physical aggression occurring in 31% to 42% of cases [12].

Factors associated with DV perpetration by older people include male sex, depression, alcohol use, low income, functional impairment, and previous violence exposure [8,13]. DV survivor status is associated with several factors including female sex, low social support, and cognitive and physical impairments [14]. A history of trauma has been linked to both being abused and perpetration of violence [14,15]. These risk factors are similar to those studied by researchers of older person abuse. Associations with being a survivor of older person abuse include disability, poor physical and mental health, cognitive impairment, and female sex [10,16,17]. Perpetrators of older person abuse are most often offspring or spouses [16-18], have mental health problems including depression and substance misuse, and are dependent on those they abuse [17,19].

IPV is often viewed as gendered [18], but this might not be the case for older person abuse. While research suggests that most survivors of IPV and older person abuse are female [7,10], some studies suggest that older male individuals are at least equally, if not more likely to experience abuse [11,20,21], and one study found that female perpetration of violence increases with age [22]. Underreporting and underdetection of the abuse of older male individuals have been noted [21,23] and occur against a broader background of underreporting of older people’s mistreatment, with estimates that 1 in 6 older people are mistreated, but only 1 in 24 cases are reported [10].

However, most of the data used for research in this area are obtained from survivor reports [9,13,24-26], which can be prone to recall bias. Thus, there is a need to explore other sources of potential information. Police records of attended DV events can provide an overview of DV by older people with the advantage of offering a relatively contemporaneous third-party perspective.

**Figure 1:**
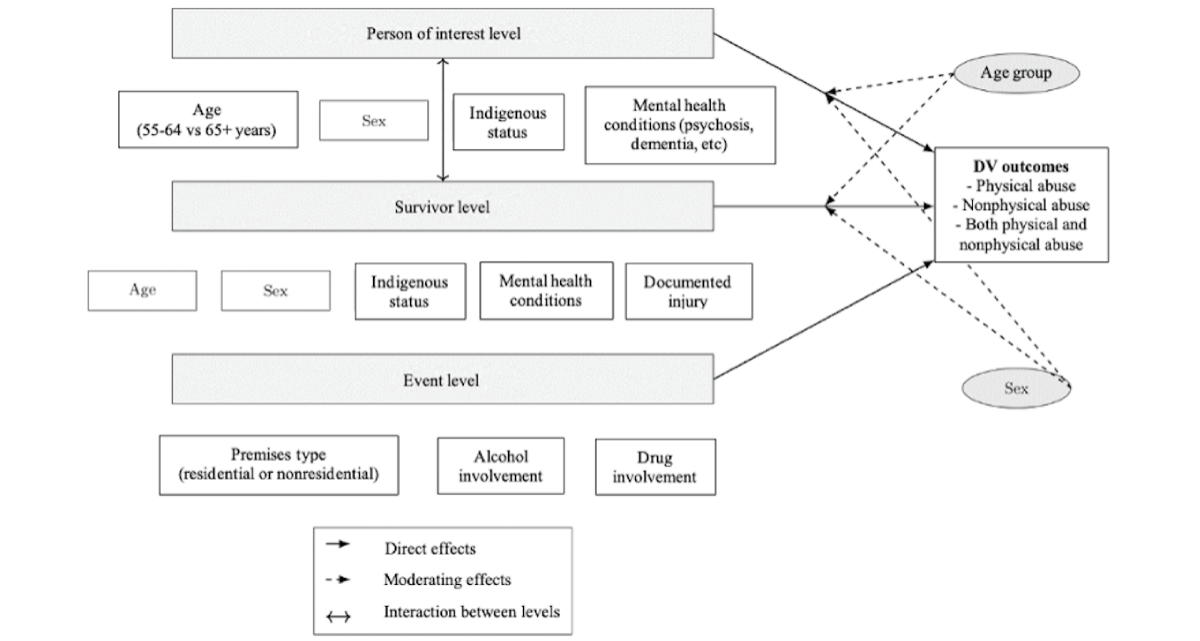
Multilevel conceptual framework for domestic violence perpetration in older adults. DV: domestic violence.

This study examined patterns of physical and nonphysical abuse among older adults using data from police-attended DV events in New South Wales. Our primary objectives were to describe the characteristics of DV events involving older persons of interest—individuals accused of or charged for perpetrating DV—and their survivors and identify potential risk factors in this understudied population. To guide our study, we developed a multilevel conceptual model (Figure 1) integrating elements from life course theory [27] and the ecological model of violence [28]. At the individual level, factors such as age-related cognitive changes affect impulse control and emotional regulation, which can lead to aggression [29]. Caregiver stress plays a role in perpetration when coping mechanisms are overwhelmed, for instance, managing behavioral symptoms of dementia [30]. The interplay between survivor and perpetrator is considered at the mesosystem level: the nature of the caregiver-recipient relationship can increase the risk of violence while feelings of threat and vulnerability can be expressed aggressively. Power dynamics can shift with aging, and expressions of control may change, for instance, from financial control to violence or from physical to verbal aggression [31]. Broader societal factors such as isolation and lack of social support for caregivers can increase violence [30], while macrosystem considerations include how violence by older people and those with cognitive impairment is construed by society, including the terminology used to describe it.

We structured the conceptual model as a 3-level hierarchical system including person of interest, survivor, and event levels. The person of interest level included characteristics of older perpetrators, including sociodemographic factors, mental health conditions (eg, dementia and psychosis), and developmental factors (eg, childhood onset disorders). The survivor level captured the dyadic context of DV, including survivor characteristics and vulnerability factors. The event level represented the immediate context of DV incidents, such as premises type and substance use involvement. We hypothesized that factors at each level exert both direct and indirect effects on DV outcomes (physical and nonphysical abuse). We hypothesized further that the perpetrator’s age group and sex may function as a cross-level moderator, which may modify the relationships between predictors and outcomes across all levels.

## Methods

### Ethical Considerations

Ethics approval for this study was obtained from the Human Research Ethics Committee of the University of New South Wales (reference: HC16558). Permission to access deidentified domestic violence events was granted by the New South Wales Police. Participants were not recruited for this study.

### Data

For the purpose of this study, an older person was defined as being aged 55 years and older based on previous DV research, which used this age as the lower age boundary [14,25,32]. The study population was taken from 492,393 police events recorded in the Computerized Operational Policing System (COPS)—a database maintained by the NSW Police Force that contains records of criminal events, including those specifically related to DV—from January 2005 to December 2016. Details regarding 64,587 events that involved either multiple persons of interest or survivors were excluded from the analysis, as it was not possible to deduce to whom the extracted mental health, survivor injury, and abuse type mention referred to (ie, if an event had 2 persons of interest and 1 extracted mental health mention for persons of interest, it is not known which person of interest the mention referred to), leaving 416,441 events for analysis, from which 221,995 events that documented the age of persons of interest were extracted. Of these, 10,708 events involved persons of interest aged 55 years and older, yielding 8247 individual persons of interest and 8629 survivors.

Each DV event in COPS has 2 primary formats: structured fields and unstructured event narratives. The structured fields contained numeric or categorical data relating to the DV event, whereas unstructured event narratives are free-text accounts that detail the circumstances surrounding the DV event. DV events were identified in COPS using specific structured fields: the offence classification being flagged as “domestic,” associated factors set as “DV-related,” and a documented domestic relationship between person of interest and survivor (including categories such as “spouse or partner,” “ex-spouse or ex-partner,” “boyfriend or girlfriend [including ex-boyfriend or ex-girlfriend, because relationship status was sometimes disputed],” “parent or guardian [including step or foster],” “child [including step or foster],” “sibling,” “other member of family [including kin],” and “caregiver”).

A text-mining approach was used to extract key information from the free-text narratives. This approach has been extensively described in a previous study [33,34]. Briefly, a set of rules based on common syntactical patterns was observed in a sample of 200 DV event narratives and identified mentions of mental illnesses, abuse types, and survivor injuries. Additionally, this involved manually constructing dictionaries containing relevant terms related to the DV event (eg, mental illness and anatomical parts). This approach identified 44 different abuse types, which included both physical and nonphysical abuse [33,34] (Multimedia Appendix 1).

The method was evaluated in a random sample of 100 narratives for the mental illness mentions with an average 92% (97.5% for persons of interest and 87.1% for survivors) precision (ie, the percentage of correctly identified mental illness mentions against the total number of identified mental illness mentions, a denominator that includes both true positives and false positives) and 90.2% and 85% precision for the abuse types and survivor injuries, respectively [33,34].

### Outcome

The primary outcome of interest in this study was to describe DV in older people and any associations with the perpetration of physical and nonphysical abuse during a DV event. Physical abuse was broadly defined to include violent actions inflicted by a person of interest on a survivor (Multimedia Appendices 1 and 2). These included life-threatening actions (eg, strangulation, stabbing, and attempting to set fire to premises), actions causing significant injury (eg, hitting with an object or weapon, kicking, and punching), actions using objects as weapons, actions involving forceful restriction (eg, blocking, throwing, and physical restraint), actions causing pain and discomfort (eg, limb twisting, scratching, and chasing), and sexual assault. Nonphysical abuse was defined as emotional (eg, threats of self-harm), social (eg, social restriction), and verbal abuse (eg, yelling profanities). Breaches of Apprehended Domestic Violence Orders (ADVO)—an order made for the protection of a person against another person with whom they have or have had a domestic relationship—were included as a separate form of abuse. “Miscellaneous or other” forms of abuse include malicious damage and unspecified (in text) abuses (eg, “assault”). Physical and nonphysical abuses that occurred in the same event were treated as separate outcomes. Injury types were grouped into 10 categories: soft tissue injuries, nonspecific wounds, soreness, fracture, periorbital bruising, stab wound, bite mark, burn mark, broken tooth, and miscellaneous (Multimedia Appendix 3).

### Explanatory Variables

Explanatory variables were classified into 3 levels based on the hierarchical nature of the data: event level (level 1), survivor level (level 2), and person of interest level (level 3). Each observation represented a unique DV event, characterized by a specific combination of persons of interest, survivor, and event date. A single survivor could have multiple events if abused by the same person of interest or by different persons of interest on different dates. A single person of interest could also be associated with multiple survivors on different dates. Individual abuse events (level 1) were nested within survivors (level 2), which were further nested within persons of interest (level 3).

Event-level variables included the following fixed field variables: premises type (residential or nonresidential), alcohol involvement in the event as assessed by police (yes or no), and drug involvement in the event as assessed by police (yes or no). Survivor-level variables encompassed age, sex (female or male), Indigenous status (non-Indigenous or Indigenous), documented injury (yes or no), and the presence or absence of psychosis, mood or anxiety disorder, personality disorder, and dementia. Person of interest–level variables included age (grouped into those aged 55-64 years and 65 years and older), sex (female or male), Indigenous status (non-Indigenous or Indigenous), and the presence or absence of psychosis, mood or anxiety disorder, personality disorder, dementia, and childhood-onset disorders, which included intellectual disability, tic disorders, and attention-deficit/hyperactivity disorder. Survivor-level and person of interest–level variables were obtained from the structured field data and the text-mined information.

### Statistical Analysis

Characteristics of persons of interest and survivors were described using summary statistics. Categorical variables were reported as frequencies and percentages, while continuous variables were summarized using means, SDs, medians, and IQRs. Differences between persons of interest and survivors were assessed using Welch *t* test for age (due to unequal variances) and chi-square tests for categorical variables. The annual proportion of DV events from 2005 to 2016 was estimated, stratified by sex and age groups. For our analysis, DV events were classified into three mutually exclusive categories: (1) physical abuse only, (2) nonphysical abuse only, and (3) both physical and nonphysical abuse occurring in the same event. This classification was implemented to accurately estimate the prevalence and predictors of each distinct type of abuse and ensure that the analysis was not confounded by the co-occurrence of multiple forms of abuse within a single event.

A 3-level mixed-effects logistic regression model was used to identify predictors of physical and nonphysical abuse while accounting for the hierarchical structure of the data. The model accounted for the clustering of individual abuse events (level 1) within survivors (level 2) and survivors within persons of interest (level 3). Variables were selected for inclusion in the regression analyses based on a literature review linking them to DV [14,15,25,35]. These included age, sex, residential status, alcohol-related event, substance use disorders, cognitive impairments, mood or anxiety disorders, psychosis, and personality factors.

The primary focus of the analysis was on estimating the fixed effects, which represented the associations between event-level, survivor-level, and person of interest–level predictors and the odds of physical and nonphysical abuse. The mixed-effects logistic regression models were systematically built. First, an empty model with only random intercepts for survivors and persons of interest was fitted to assess the baseline variability in abuse outcomes attributable to differences across persons of interest and survivors. Second, event-level, survivor-level, and perpetrator-level factors were included in the multivariable model (full model) to determine adjusted estimates. The initial multivariable model included all variables of interest regardless of statistical significance from the univariable analyses. Variables with a *P* value of >.20 from the univariable analyses were individually dropped, and the Akaike information criterion was used to compare the initial multivariable model and the model without these variables. The model resulting in a smaller Akaike information criterion was considered as the better-fitting model. Adjusted odds ratios (aORs) with 95% CIs were reported as measures of association. Statistical significance was assessed using a 2-tailed *P* value threshold of .05. We examined whether the associations between explanatory variables and outcomes of interest differed significantly between person of interest age groups (55-64 years and 65 years and older) by conducting a stratified analysis by person of interest age groups.

Random effects parameters were estimated to quantify the variability in abuse outcomes attributable to the hierarchical structure of the data. The intraclass correlation coefficient was calculated to determine the proportion of the total variance in abuse events that was due to differences across persons of interest and survivors. The proportional change in variance was computed to assess the reduction in unexplained variance after adding predictors to the models. Multiple imputation by chained equations was used to address the missing data, which was determined to be missing at random. In total, 100 datasets were imputed. All analyses were conducted with Stata statistical software (version 18; StataCorp LLC).

## Results

### Characteristics of Police-Attended DV Events

Our analysis included 8247 persons of interest and 8629 survivors involved in 10,708 DV events (Multimedia Appendices 4 and 5). Male individuals constituted 81.4% (n=6710) of persons of interest, and female individuals comprised 73.9% (n=6375) of survivors (Table 1). This sex difference was consistent across age groups. Among persons of interest aged 55-64 years, 80.9% (n=4752) were male individuals, and 19% (n=1172) were female individuals. In the 65+-year age group, 82.5% (n=1958) of persons of interest were male individuals, and 17.5% (n=415) were female individuals. Persons of interest were typically older than survivors, with a median age of 60 (IQR 57-65) years for persons of interest compared to 50 (IQR 37-60) years for survivors.

**Table 1:** Characteristics of persons of interest and survivors of domestic violence events.

			Persons of interest (n=8247)		Survivors (n=8629)		*P* value^a^	
**Age (years)**								<.001^b^
	Mean (SD)	62 (7.0)		49 (16.9)				
	Median (IQR)	60 (57-65)		50 (37-60)				
**Sex, n (%)**								<.001
	Female	1532 (18.6)		6375 (73.9)				
	Male	6710 (81.4)		2243 (26)				
**Indigenous status, n (%)**								.19
	Indigenous	303 (3.7)		285 (3.3)				
	Non-Indigenous	7944 (96.3)		8344 (96.7)				
**Dementia, n (%)**								
	Present	191 (2.3)		104 (1.2)		<.001		
**Psychosis, n (%)**								
	Present	142 (1.7)		87 (1)		<.001		
**Mood or anxiety disorder**								
	Present	56 (0.7)		47 (0.5)		.13		
**Personality disorder, n (%)**								
	Present	28 (0.3)		13 (0.2)		.02		
**Childhood-onset disorder, n (%)**								
	Present	97 (1.2)		92 (1.1)		.51		
**Alcohol and substance use disorder, n (%)**								
	Present	200 (2.4)		65 (0.8)		<.001		

^a^*P* values for categorical variables calculated using the chi-square test of independence.

^b^*P* value for age calculated using the Welch *t* test (*t* test for unequal variances) due to the large difference in SDs between the 2 groups.

Overall, persons of interest had higher proportions of mental health conditions compared with survivors. Dementia was observed in 2.3% (n=191) of persons of interest compared to 1.2% (n=104) of survivors (difference: n=87, 1.1%; *P*<.001). Psychosis showed a similar pattern, with a prevalence of 1.7% (n=142) in persons of interest versus 1% (n=87) in survivors (difference: n=55, 0.7%; *P*<.001). The largest difference was observed in alcohol and substance use disorders: 2.4% (n=200) of persons of interest had these disorders compared to 0.8% (n=65) of survivors (difference: n=135, 1.6%; *P*<.001). Personality disorders were slightly more common in persons of interest (n=28, 0.3%) than in survivors (n=13, 0.2%), with a small difference (n=15, 0.1%; *P*=.01).

Most (n=7441, 64.5%) events involved male persons of interest and female survivors, and the majority (n=9399, 87.8%) occurred in residential settings (Table 2). Persons of interest aged 55-64 years had a higher proportion of survivors who were ex-spouses or ex-partners (n=890, 11.5% vs n=240, 8.1%), and fewer survivors who were spouses (n=2191, 28.3% vs n=1126, 37.8%) compared with those persons of interest aged 65 years and older. Most of the charges were related to physical violence, predominantly assaults. Homicide was uncommon and more prevalent in people aged 65 years and older, as was firearm use (n=11, 0.4%). In most events (n=6636, 62%), no weapon was recorded.

**Table 2:** Characteristics of police-attended domestic violence (DV) events in New South Wales, stratified by age.

Characteristics		All DV events (N=10,708), n (%)	DV events from persons of interest aged 55-64 years (n=7732), n (%)	DV events from persons of interest aged 65+ years (n=2976), n (%)
**Paired sex (person of interest-survivor)**				
	Male-female	7441 (64.5)	5347 (69.2)	2094 (70.4)
	Female-female	752 (7)	539 (7)	213 (7.2)
	Male-male	1486 (13.9)	1077 (13.9)	409 (13.7)
	Female-male	1013 (9.5)	757 (9.8)	256 (8.6)
**Person of interest relationship with survivor**				
	Boyfriend or girlfriend (including ex)	704 (6.6)	597 (7.7)	107 (3.6)
	Spouse or partner	3317 (31)	2191 (28.3)	1126 (37.8)
	Ex-spouse or partner	1130 (10.6)	890 (11.5)	240 (8.1)
	Parent or guardian of survivor	951 (8.9)	679 (8.8)	272 (9.1)
	Child (including step or foster child) of survivor	273 (2.6)	213 (2.8)	60 (2)
	Sibling	227 (2.1)	170 (2.2)	57 (1.9)
	Other member of family	1017 (9.5)	695 (9)	322 (10.8)
	Unknown or unrecorded	3089 (28.9)	2297 (29.7)	792 (26.6)
**Event premises**				
	Residential	9399 (87.8)	6762 (87.5)	2637 (88.6)
	Nonresidential	1309 (12.2)	970 (12.6)	339 (11.4)
**Events associated with drugs**				
	Present	30 (0.3)	27 (0.3)	3 (0.1)
**Events associated with alcohol**				
	Present	3631 (33.9)	2845 (36.8)	786 (26.4)
**Alleged offence associated with event**				
	Homicide	28 (0.3)	16 (0.2)	12 (0.4)
	Assault	7213 (67.4)	5085 (65.8)	2128 (71.5)
	ADVO^a^ breach	2203 (20.6)	1711 (22.1)	492 (16.5)
	Other offense against person	1779 (16.6)	1273 (16.5)	506 (17)
	Malicious damage	734 (6.9)	593 (7.7)	141 (4.7)
**Survivor injury documented^b^**				
	Soft tissue injury	2304 (21.5)	1660 (21.5)	644 (21.6)
	Wound (nonspecific)	1468 (13.7)	1089 (14.1)	379 (12.7)
	Soreness	641 (6)	473 (6.1)	168 (5.7)
	Fracture	318 (3)	228 (3)	90 (3)
	Periorbital bruising	48 (0.4)	22 (0.3)	26 (0.9)
	Stab wound	34 (0.3)	25 (0.3)	9 (0.3)
	Bite or burn mark	27 (0.3)	21 (0.3)	6 (0.2)
	Broken tooth	14 (0.1)	12 (0.2)	2 (0.1)
	Other	416 (3.9)	303 (3.9)	113 (3.8)
	No injuries reported	6,931 (64.7)	4982 (64.4)	1949 (65.5)
**Weapon use**				
	Sharp instrument	308 (2.9)	218 (2.8)	90 (3)
	Blunt instrument	98 (0.9)	68 (0.9)	30 (1)
	Firearm-related	23 (0.2)	12 (0.2)	11 (0.4)
	Weapon unrecorded	6636 (62)	4657 (60.2)	1979 (66.5)
**Abuse type**				
	Physical abuse only	3651 (34.1)	2610 (33.8)	1041 (35)
	Nonphysical abuse only	1286 (12)	918 (11.9)	368 (12.4)
	Physical and nonphysical abuse	2452 (22.9)	1771 (22.9)	681 (22.9)
	No abuse event recorded	3319 (31)	2433 (31.5)	886 (29.8)

^a^ADVO: Apprehended Domestic Violence Order.

^b^Subcategories are not mutually exclusive. Multiple injuries and alleged offences could be recorded in each event; hence, subcategories exceed the total number of events.

At least 1 type of abuse was recorded in 69% (n=7389) of event narratives. Physical abuse events were the most common type of abuse (n=3651, 34.1%), followed by combined physical and nonphysical abuse (n=2452, 22.9%) and nonphysical abuse events (n=1286, 12%).

### Trends in Police-Attended DV Events From 2005 to 2016

Over the period 2005-2016, the proportions of physical and nonphysical abuse events involving persons of interest aged 55-64 years (“younger” group) and those aged 65 years and older (“older” group) remained relatively stable over time for both sexes. However, female persons of interest aged 65 years and older showed higher and more variable proportions of physical abuse events compared to their male counterparts (Multimedia Appendix 6).

Female persons of interest in the older group had higher proportions of physical abuse and nonphysical abuse compared to those in the younger group (Multimedia Appendix 7). They also had smaller proportions of abuse events related to combined physical and nonphysical abuse events, breach of ADVOs, and other events. The proportion of abuse types was similar in male counterparts aged 55-64 years and 65 years and older, except for proportionally fewer breaches of ADVOs in the older group.

### Multilevel Associations of Physical and Nonphysical Abuse

The multilevel analysis revealed several factors associated with physical abuse, nonphysical abuse, and both physical and nonphysical abuse (Tables 3 and 4). Alcohol-related events (aOR 1.04, 95% CI 1.02-1.06), survivor dementia (aOR 1.10, 95% CI 1.01-1.21), and persons of interest aged 65 years and older (aOR 1.02, 95% CI 1.00-1.05) were associated with higher odds of physical abuse. Conversely, persons of interest with substance use disorders had a lower odds of physical abuse (aOR 0.94, 95% CI 0.89-0.99). The inclusion of predictors in the model for the physical abuse outcome explained 69.40% of the person of interest–level variance compared to the null model.

For nonphysical abuse, male persons of interest had higher odds of perpetration (aOR 1.02, 95% CI 1.01-1.04), as did Indigenous persons of interest (aOR 1.04, 95% CI 1.00-1.08). Survivors with dementia had lower odds of experiencing nonphysical abuse (aOR 0.93, 95% CI 0.87-0.99). Indigenous survivors showed decreased odds of nonphysical abuse (aOR 0.96, 95% CI 0.92-1.00), albeit at the threshold of statistical significance. The predictors in the model for nonphysical abuse explained 96.50% of the survivor-level variance compared to the null model.

Events at residential premises were associated with higher odds of both (or co-occurring) physical and nonphysical abuse (aOR 1.05, 95% CI 1.03-1.08), as were alcohol-related events (aOR 1.03, 95% CI 1.02-1.05). Male survivors had lower odds of experiencing both physical and nonphysical abuse (aOR 0.97, 95% CI 0.95-0.99). Indigenous persons of interest had lower odds of perpetrating both physical and nonphysical abuse (aOR 0.94, 95% CI 0.90-0.99), while persons of interest with dementia (aOR 1.07, 95% CI 1.01-1.13) or a substance use disorder (aOR 1.13, 95% CI 1.08-1.19) had higher odds. The predictors in the model for both physical and nonphysical abuse explained 33.35% of the survivor-level variance compared to the null model.

In examining the consistency of findings across the 3 models (physical abuse only, nonphysical abuse only, and both physical and nonphysical abuse), several patterns emerged. Alcohol-related events were consistently associated with higher odds of physical abuse and both physical and nonphysical abuse. Person of interest with substance use disorder showed opposite effects: lower odds of physical abuse but higher odds of both physical and nonphysical abuse. Dementia in survivors was associated with higher odds of physical abuse but lower odds of nonphysical abuse, while person of interest with dementia was associated with higher odds of both physical and nonphysical abuse. Indigenous status showed varying associations: Indigenous survivors had lower odds of nonphysical abuse, while Indigenous persons of interest had higher odds of nonphysical abuse but lower odds of both physical and nonphysical abuse.

**Table 3:** Multilevel predictors of physical and nonphysical abuse.

Characteristics					Full model for physical abuse only				Full model for nonphysical abuse only				Full model for both physical and nonphysical abuse		
					aOR^a^ (95% CI)		*P* value		aOR (95% CI)		*P* value		aOR (95% CI)		*P* value
**Event level**															
	**Premises**														
			Nonresidential	1.0 (reference^b^)		—^c^		1.0 (reference)		—		1.0 (reference)		—	
			Residential	1.01 (0.99-1.04)		.34		1.01 (0.99-1.02)		.57		1.05 (1.03-1.08)		<.001	
	**Alcohol-related event**														
			No	1.0 (reference)		—		1.0 (reference)		—		1.0 (reference)		—	
			Yes	1.04 (1.02-1.06)		<.001		1.01 (0.99-1.02)		.36		1.03 (1.02-1.05)		<.001	
	**Drug-related-event**														
			No	1.0 (reference)		—		1.0 (reference)		—		1.0 (reference)		—	
			Yes	1.01 (0.85-1.19)		.92		0.92 (0.82-1.03)		.15		0.96 (0.83-1.11)		.58	
**Survivor level**															
	**Survivor injury documented**														
			No	1.0 (reference)		—		1.0 (reference)		—		1.0 (reference)		—	
			Yes	1.16 (1.14-1.18)		<.001		0.93 (0.92-0.94)		<.001		1.08 (1.07-1.10)		<.001	
	**Survivor sex**														
			Female	1.0 (reference)		—		1.0 (reference)		—		1.0 (reference)		—	
			Male	1.01 (0.99-1.04)		.29		1.00 (0.98-1.02)		.98		0.97 (0.95-0.99)		.003	
	**Survivor age (years)**														
		Per-year increase in age		0.99 (0.99 – 0.99)		.04		1.00 (1.00-1.00)		.03		—		—	
	**Indigenous status (survivor)**														
			Non-Indigenous	1.0 (reference)		—		1.0 (reference)		—		1.0 (reference)		—	
			Indigenous	1.05 (0.99-1.12)		.11		0.96 (0.92-1.00)		.05		1.03 (0.98-1.09)		.28	
	**Psychosis (survivor)**														
			Not present	1.0 (reference)		—		1.0 (reference)		—		1.0 (reference)		—	
			Present	1.00 (0.91-1.10)		.95		1.02 (0.96-1.09)		.47		1.00 (0.92-1.09)		.96	
	**Dementia (survivor)**														
			Not present	1.0 (reference)		—		1.0 (reference)		—		1.0 (reference)		—	
			Present	1.10 (1.01-1.21)		.03		0.93 (0.87-0.99)		.02		0.95 (0.88-1.03)		.23	
	**Childhood-onset disorder (survivor)**														
			Not present	1.0 (reference)		—		1.0 (reference)		—		1.0 (reference)		—	
			Present	0.97 (0.89-1.07)		.54		1.00 (0.94-1.07)		.93		0.96 (0.89-1.05)		.39	
	**Self-harm (survivor)**														
			Not present	1.0 (reference)		—		1.0 (reference)		—		1.0 (reference)		—	
			Present	1.04 (0.89-1.21)		.62		1.02 (0.91-1.13)		.76		0.93 (0.81-1.06)		.29	
	**Substance use disorder (survivor)**														
			Not present	1.0 (reference)		—		1.0 (reference)		—		1.0 (reference)		—	
			Present	0.94 (0.84-1.04)		.24		1.06 (0.99-1.14)		.10		1.00 (0.91-1.10)		.98	
	**Personality disorder (survivor)**														
			Not present	1.0 (reference)		—		1.0 (reference)		—		1.0 (reference)		—	
			Present	1.02 (0.80-1.30)		.85		1.05 (0.89-1.25)		.53		1.00 (0.80-1.23)		.97	
**Person of interest level**															
	**Person of interest** **sex**														
			Female	1.0 (reference)		—		1.0 (reference)		—		1.0 (reference)		—	
			Male	0.98 (0.95-1.00)		.07		1.02 (1.01-1.04)		.01		0.98 (0.96-1.01)		.14	
	**Person of interest** **age **														
			55-64	1.0 (reference)		—		1.0 (reference)		—		1.0 (reference)		—	
			65+	1.02 (1.00-1.05)		.03		1.00 (0.98-1.01)		.91		1.00 (0.98-1.02)		.92	
	**Indigenous status (person of interest)**														
			Non-Indigenous	1.0 (reference)		—		1.0 (reference)		—		1.0 (reference)		—	
			Indigenous	1.01 (0.95-1.07)		.74		1.04 (1.00-1.08)		.04		0.94 (0.90-0.99)		.02	
	**Psychosis (person of interest)**														
			Not present	1.0 (reference)		—		1.0 (reference)		—		1.0 (reference)		—	
			Present	1.00 (0.93-1.08)		>.99		1.02 (0.97-1.08)		.36		1.06 (0.99-1.13)		.09	
	**Dementia (person of interest)**														
			Not present	1.0 (reference)		—		1.0 (reference)		—		1.0 (reference)		—	
			Present	0.98 (0.92-1.04)		.52		1.02 (0.97-1.06)		.50		1.07 (1.01-1.13)		.02	
	**Childhood-onset disorder (person of interest)**														
			Not present	1.0 (reference)		—		1.0 (reference)		—		1.0 (reference)		—	
			Present	1.00 (0.92-1.09)		.96		1.03 (0.97-1.10)		.26		1.06 (0.98-1.15)		.13	
	**Self-harm (person of interest)**														
			Not present	1.0 (reference)		—		1.0 (reference)		—		1.0 (reference)		—	
			Present	1.10 (0.97-1.24)		.14		0.98 (0.90-1.06)		.57		0.98 (0.88-1.10)		.77	
	**Substance use disorder (person of interest)**														
			Not present	1.0 (reference)		—		1.0 (reference)		—		1.0 (reference)		—	
			Present	0.94 (0.89-0.99)		.02		1.00 (0.96-1.04)		.92		1.13 (1.08-1.19)		<.001	
	**Personality disorder (person of interest)**														
			Not present	1.0 (reference)		—		1.0 (reference)		—		1.0 (reference)		—	
			Present	0.89 (0.74-1.06)		.19		1.00 (0.88-1.13)		.99		1.02 (0.87-1.19)		.81	

^a^aOR: adjusted odds ratio.

^b^Reference category indicates the baseline group against which the other category or categories are compared for the specific abuse category.

^c^Not applicable.

**Table 4:** Random effects parameters.

Parameters			Full model for physical abuse only		Full model for nonphysical abuse only		Full model for both physical and nonphysical abuse
**Persons of interest**							
	Variance	0.003		0.01		0.06	
	ICC^a^ (%)	1.45		7.51		3.32	
	PCV^b^ (%)	69.40		–17.27		6.23	
**Survivors nested within** **persons of interest**							
	Variance	0.02		Negligible		0.05	
	ICC (%)	9.24		0		3.16	
	PCV (%)	4.45		96.50		33.35	

^a^ICC: intraclass correlation coefficient.

^b^PCV: proportional change in variance.

### Stratified Multilevel Analysis by Person of Interest Age Groups

#### Factors With the Same Direction of Effect Among Both Age Groups

In the stratified analysis by person of interest age (Multimedia Appendices 8-10), several factors showed consistent directions of effect for both person of interest age groups across all 3 abuse outcomes. For physical abuse, residential premises increased odds in both age groups (55-64 years: aOR 1.38, 95% CI 1.17-1.63 and 65 years and older: aOR 1.33, 95% CI 1.02-1.74). Person of interest sex (female) showed an increase in odds of physical abuse for those aged 55-64 years (aOR 1.22, 95% CI 1.04-1.44) but lower odds of nonphysical abuse (aOR 0.72, 95% CI 0.57-0.90). Among female persons of interest, female survivors had higher odds of experiencing physical abuse compared to male survivors (aOR 1.39, 95% CI 1.09-1.78); however, among male persons of interest, female survivors had significantly higher odds of experiencing both physical and nonphysical abuse (aOR 1.27, 95% CI 1.09-1.48). Male-perpetrated physical abuse was more likely in residential premises (aOR 1.42, 95% CI 1.21-1.66); however, no such difference was observed for female-perpetrated abuse. There were also no differences between survivor age and abuse type among male or female persons of interest.

Documented survivor injury was associated with physical abuse in both groups (55-64 years: aOR 3.28, 95% CI 2.88-3.75 and 65 years and older: aOR 2.86, 95% CI 2.35-3.48). A similar trend was observed for both physical and nonphysical abuse; however, substance use disorder in persons of interest was an additional risk factor in both groups (55-64 years: aOR 1.87, 95% CI 1.36-2.56 and 65 years and older: aOR 1.98, 95% CI 1.13-3.45). In the case of nonphysical abuse, documented survivor injury was associated with lower odds in both age groups (55-64 years: aOR 0.44, 95% CI 0.37-0.53 and 65 years and older: aOR 0.47, 95% CI 0.35-0.62).

#### Factors With Opposite Direction of Effect Among Both Age Groups

Some factors showed opposite directions of effect between the 2 person of interest age groups. For physical abuse, the Indigenous status of person of interest decreased odds for the 55- to 64-year group (aOR 0.68, 95% CI 0.49-0.95) but increased odds for the 65+-year group (aOR 1.60, 95% CI 0.71-3.58), although the latter was not statistically significant. A similar pattern was observed for both physical and nonphysical abuse regarding the Indigenous status of person of interest (55-64 years: aOR 0.62, 95% CI 0.44-0.88 and 65 years and older: aOR 1.34, 95% CI 0.58-3.08).

For nonphysical abuse, alcohol-related events were associated with decreased odds for the 55- to 64-year group (aOR 0.91, 95% CI 0.78-1.08) but with significantly higher odds for the 65+-year group (aOR 1.68, 95% CI 1.28-2.20). Substance use disorder in survivors was associated with increased odds for the 55- to 64-year group (aOR 2.15, 95% CI 1.03-4.48) but decreased odds for the 65+-year group (aOR 0.82, 95% CI 0.16-4.24), though this was not statistically significant for the older group.

Dementia in persons of interest was associated with significantly higher odds of both physical and nonphysical abuse in the 65+-year group (aOR 1.73, 95% CI 1.19-2.53), but not in the 55- to 64-year group (aOR 0.81, 95% CI 0.31-2.14).

## Discussion

### Main Findings

This study examined 10,708 police-attended DV events involving 8247 persons of interest aged 55 years and older and 8629 survivors in New South Wales, Australia, from 2005 to 2016. Consistent with the literature on DV across the age spectrum [4] including older people [7,14], most persons of interest were male, and most of the survivors were female. Initially, descriptive analyses suggested a higher and more variable proportion of physical abuse events among female persons of interest aged 65 years and older. However, after adjusting for confounders and accounting for data clustering, female persons of interest aged 55-64 years and 65 years and older showed similarly elevated odds of physical abuse compared to male persons of interest. This insight aligns with existing research that indicates that female-perpetrated abuse increases with increasing survivor age [22] and that older male individuals are more likely to report exposure to violence [13]. Female perpetration of DV is still underresearched, and this age-group association might reflect the stress of caregiver responsibilities or shifts in power relationships due to survivor dependency.

The association between abuse type and location differed by perpetrator sex, with male-perpetrated physical abuse more likely in residential settings, a pattern not observed for female perpetrators. Furthermore, while the overall odds of physical abuse decreased with survivor age and nonphysical abuse increased, these age-related patterns in exposure to violence were no longer evident after stratification by perpetrator sex. Notably, substance use disorder was consistently associated with higher odds of abuse across both person of interest age groups (55-64 and 65+ years). However, dementia and mental disorders typically diagnosed in childhood were associated with higher odds of both physical and nonphysical abuse only in the 65+-year age group. These findings suggest age-specific risk factors for DV perpetration in later life, with cognitive impairment and long-standing mental health issues emerging as significant contributors with increasing age. However, as these data are cross-sectional and observational in nature, an interplay of factors or unobserved factors might also account for these findings, and further studies are required.

At the person of interest level, there were higher odds of physical abuse in the group aged 65 years and older compared with the younger group, which might reflect the inclusion of physical abuse perpetrated by people with dementia in this study or abuse perpetrated by aging caregivers. Physical abuse and combined physical and nonphysical abuse events were more common than nonphysical abuse events. This might initially appear to contradict the typical picture of DV in older people, which is declining physical abuse with age [14,36], and could be because these data only cover events where police were called, which is more likely to occur if a person is concerned about physical safety. The decision to call the police in situations of DV is complex [32] and made more complex in intimate relationships where the perpetrator has a mental health condition that the survivor believes contributes to their behavior, with survivors reporting a sense of obligation to stay with the perpetrator [25]. The obligation to stay with the perpetrator can also occur in other relationships, such as adult children who care for their parents. One study found that police notifications for DV decreased with survivor age [29], possibly because the authors limited their data to events where survivors had contacted the police, whereas these data included all police callouts.

Compared with survivors, mental health conditions were more common in persons of interest who had a greater proportion of dementia (n=191, 2.3% vs n=104, 1.2%), psychosis (n=142, 1.7% vs n=87, 1%), and alcohol and substance use disorder (n=200, 2.4% vs n=65, 0.8%), consistent with other research [9,14]. Alcohol may have played a situational role in events involving physical abuse compared to nonphysical abuse, while persons of interest with substance use disorders (which include alcohol use disorders) increased the odds of DV perpetration involving both physical and nonphysical abuse when compared with physical-only and nonphysical-only abuse. Alcohol use disorders have been consistently associated with DV in later age [7,9,14,15], and the role of acute alcohol consumption has also been noted in IPV research [8,25]. Alcohol tolerance decreases with age, leading to higher and more prolonged alcohol levels compared with younger people [34]. Acute intoxication impairs most cognitive functions [35], including judgment and impulse control, and may be pertinent for new-onset DV in older people who consume alcohol and are unaware of their reduced tolerance. Substance use disorders in older people are associated with cognitive impairment, particularly visuoperceptual and executive dysfunction [35], which could explain the association with the commission of violence.

The effects of dementia were exerted at both a survivor and perpetrator level, increasing the odds of solely physical and solely nonphysical exposure to violence compared with the combined abuse group. The finding that dementia was associated with increased exposure to violence is consistent with other studies of DV survivors with dementia [36,37]. Exposure to violence of people with dementia has been viewed through the prism of caregiver stress [15,38], and this study adds to the literature, finding that the odds of such exposure to violence are increased for physical abuse but decreased for nonphysical abuse types. The role of caregiver stress in the perpetration of abuse is not linear, and there are likely mediating factors. The results of this study also found that dementia increased the odds of being a person of interest in events where combined physical and nonphysical abuse occurred, consistent with the findings of high levels of aggression in people with dementia [12,39]. This complex relationship between dementia and violent behavior suggests the need for further examination of specific characteristics that are associated with exposure to violence and perpetration of violence in this group.

A greater proportion of homicide (n=12, 0.4% vs n=16, 0.2%) and assault charges (n=2128, 71.5% vs n=5085, 65.8%) were laid against persons of interest in the older group. The older group also had a greater proportion of firearm use (n=11, 0.4% vs n=12, 0.2%), a finding that requires further examination. Weapons might be used by older people to compensate for decreased physical strength but have a greater propensity for resulting in injury and death, particularly if wielded against a spouse who is similarly aged.

### Future Research and Policy Directions

This study has demonstrated shared and separate factors involved in the perpetration of physical and nonphysical abuse in older age. Future research is required into examining this association and determining the salience of these factors for DV that emerges in later life, as opposed to younger onset DV. For instance, Policastro et al [9] have suggested that new onset DV in older people may be more likely to be associated with mental illness and dementia, whereas DV “grown old” usually presents with emotional abuse.

Further, traditional theoretical frameworks underpinning the commission of violence in younger people may require adaptation for their application to older people. DV and, more recently, abuse of older people are viewed through a lens of power and control [7,19], while abuse of older people also acknowledges the role of caregiver stress [15]. These frameworks will need to accommodate the role of biological aging on alcohol tolerance and cognition as well as changes in cognition associated with the onset of neurological disorders. Conversely, rather than viewing aggression in people with dementia solely as a symptom of the condition, a consideration of premorbid relationship dynamics and scrutiny of premorbid behaviors is required to determine if the dementia is an exculpatory factor. The role of premorbid personality was demonstrated in a study where caregivers of 99 people with dementia who displayed aggressive behavior were interviewed. In 3% of cases, the behaviors were similar to their premorbid state; in 43% of cases, it was an exaggeration of premorbid behavior; and in 53% of people, the aggression was different from their premorbid behavior [39].

Qualitative studies are recommended to understand the dynamics of relationships between the perpetrator and survivor, which may be influenced by caring responsibilities, frailty, and disability, in addition to earning constraints and social and cultural factors. The data also point to the need for further research to understand older female individuals who perpetrate violence, a particularly neglected group.

From a policy perspective, this research underlines the need for a multipronged approach to target substance use in older people, including education concerning safe levels of alcohol consumption in older age provided at an individual level through health providers and at a population level through government advertising campaigns with focus group evaluation of their success. This is particularly pertinent as the generation of adults born in the “baby boomer” generation because this generation has comparatively high use of alcohol and drugs compared to previous older generations [40].

The findings of this study have implications for clinicians who treat people with dementia, as dementia increases the odds of being both a survivor and a perpetrator of abuse. Health professionals who treat older people in primary and secondary care require education to recognize signs of nonaccidental injury in older people with dementia and to specifically enquire about the patient’s alcohol use, signs of aggression, agitation, and psychosis, particularly if they reside with another older person who might be at risk of serious injury or death from violence. This could be undertaken by professional specialty medical and nursing societies for emergency medicine, geriatrics, and old age psychiatry.

### Strengths and Limitations

A strength of this study is that it did not rely on survivor recall of events, which could be contaminated by recall bias, although qualitative data would be useful for a greater understanding of the reasons why police are called. This is one of the largest datasets of older DV covering both male and female persons of interest and survivors. Yon et al [41] combined 2 cross-sectional datasets to obtain 6971 male and female survivors aged 60 years and older, Zink et al [36] looked at 995 female survivors aged 55 years and older, while Yan and Chan [13] examined 937 cases who were aged 60 years and older for IPV, including 540 male individuals.

This dataset is unable to cover the array of interconnected factors that influence DV, including cultural and environmental factors, such as resourcing, relationship factors, and individual factors, such as education and income level. These issues are multifaceted and, because they are strongly affected by cultural norms [42], these results may not be generalizable to all cultures and should be interpreted in conjunction with culture-specific qualitative data.

In addition, it is possible that older adults might be hesitant to report DV events to the police for several reasons, and as such, the number of police DV events involving older perpetrators and survivors could be underreported. Older people might not recognize abusive behaviors often focusing on intentional physical abuse while overlooking other forms, such as neglect, social, or financial abuse [43,44]. For example, Acierno et al [44] mentioned that financial abuse was less likely to be reported to authorities when perpetrated by a family member compared to similar acts by strangers. Furthermore, relationships between elder survivors and perpetrators such as caregivers or adult children may discourage reporting due to feelings of guilt and the desire to protect family members from legal consequences [45,46]. Finally, elder survivors may be unaware of the appropriate authority or available services to disclose their abuse to [47].

The use of police data required a notification, and deciding to call the police in cases of DV can be complex, depending on the relationship between the survivor and the person of interest. Further, concerns of police bias against people due to their race, socioeconomic status, sexuality, or past encounters can dissuade people from seeking police assistance [48]. Cases where the person experiencing abuse was reluctant to tell the police about the abuse (eg, due to resolution of the disagreement, fear of the person of interest, concerns about police bias, or stigma) and where there was no obvious sign of injury or disturbance may have been relegated to the “no abuse event recorded” category, such that underreporting of abuse types could occur, which is particularly salient for older people, who are more reluctant to disclose abuse if they are dependent on the perpetrator [32] and because of generational norms [49]. It is also likely that police would record only observable or reported survivor injuries, leading to potential underreporting in people with dementia who were unable to communicate how and where they were injured if it was not obviously visible, and the occurrence of nonphysical abuse types.

Police officers are not experts in diagnosing mental illnesses and rely on the testimony of witnesses at the DV event and on the self-reports of persons of interest and survivors. Therefore, the accuracy of mental illness mentions within police narratives remains unclear, considering that the purpose of collecting such data is not for research, and any interpretation of our findings should be taken with caution [50]. Any misclassification of mental health status for persons of interest (or survivors) in the narratives could potentially produce wrong conclusions for specific conditions (eg, dementia) in the DV setting. Underreporting of dementia in persons of interest might underestimate its association with physical and nonphysical abuse, affecting the respective aORs in Table 3 (eg, aOR 1.07, 95% CI 1.01-1.13 for dementia in persons of interest for combined physical and nonphysical abuse). On the other hand, overreporting based on subjective observations in the police narratives could inflate these associations for conditions like dementia, which require clinical assessment, affecting the precision of our aORs, potentially leading to wider CIs or effect sizes. To address this, future research could focus on linking police records with clinical datasets to validate mental health mentions, enabling the application of correction methods to account for diagnostic uncertainty [51].

### Conclusions

This study suggests that physical and nonphysical DV persists in later life. Alcohol and dementia increase the likelihood of perpetration, and screening should target these factors. Instead of reactively treating aggression that arises in the context of dementia, clinicians should regularly evaluate the risk of violence and exposure to violence in patients with dementia and educate caregivers about potential violent behavior early in the course of the condition. This would enable the provision of information on services that can be used to assist in the management of these behaviors. Additionally, clinicians require education to be alert for nonaccidental injury in people with dementia. Pediatric services have guidelines and are aware of red flags for suspected child abuse and neglect [52], which can inform the development of similar protocols for geriatric medical services.

## Appendix

Multimedia Appendix 1 Abuse categories and abuse types.

Multimedia Appendix 2 Description of the extracted abuse types.

Multimedia Appendix 3 Categories used to group survivor injury types derived from text mining of police narratives.

Multimedia Appendix 4 Yearly breakdown of total police-reported events by sex (female and male) for persons of interest aged 55-64 years (2005-2016).

Multimedia Appendix 5 Yearly breakdown of total police-reported events by sex (female and male) for persons of interest aged 65+ years (2005-2016).

Multimedia Appendix 6 Proportion of physical and nonphysical abuse incidents involving female and male persons of interest aged 55-64 and 65+ years, annually from 2005 to 2016.

Multimedia Appendix 7 Abuse types police-attended domestic violence events in New South Wales from 2005 to 2016, by sex and age group.

Multimedia Appendix 8 Predictors of physical abuse, stratified by person of interest age groups.

Multimedia Appendix 9 Predictors of both physical and nonphysical abuse, stratified by person of interest age groups.

Multimedia Appendix 10 Predictors of nonphysical abuse, stratified by person of interest age groups.

## Abbreviations

ADVO: Apprehended Domestic Violence Order
aOR: adjusted odds ratio
BPSD: behavioral and psychological symptoms of dementia
COPS: Computerized Operational Policing System
DV: domestic violence
IPV: intimate partner violence

## Data Availability

Due to the sensitive nature of police documents, the data and materials are not available for replication purposes.
