# Supplementary material for: Older Perpetrators of Domestic Violence: Mixed-Effects Logistic Regression Analysis of Police Records
Source: JMIR Aging. 2025 Sep 29;8:e75993. doi: 10.2196/75993 (PMC12519033; doi:10.2196/75993)
Supplement: Multimedia Appendix 1 [file aging_v8i1e75993_app1.docx]

| **Category** | **Abuse Type** |
| --- | --- |
| **Physical Abuse** | Assault (unspecified), biting, blocking, choking, ordered dog attack, dragging, elbowing, attempting to set fire to premises, gagging, grabbing, hair pulling, headbutting, head locking, kicking, kneeing, physical restraining, pulling, punching, pushing, scratching, shaking, slapping, spitting, stabbing, victim being thrown around, limb twisting, attempt to harm a victim with an object or weapon, and hitting the victim with an object or weapon.  Sexual assault (e.g., rape). |
| **Non-physical abuses** | Intimidation (via body language) or stating explicit threat(s) to physically harm, sexually assault, and self-harm if the victim does not comply.  Self-harming when the victim does not comply, yelling profanities, and other emotional/verbal abuse.  Stalking, harassment, and forced entry.  Financial control (e.g., no access to credit card).  Social restriction and prevent/limit child access. |
| **Apprehended Domestic Order Breach (ADVO)** | Apprehended Domestic Violence Order breach. |
| **Miscellaneous** | Property damage (ranging from breaking an item to causing damage to a house or vehicle), chasing, lunging, other, and possession of personal effects (e.g., phone and car keys). |
