# Supplementary material for: Older Perpetrators of Domestic Violence: Mixed-Effects Logistic Regression Analysis of Police Records
Source: JMIR Aging. 2025 Sep 29;8:e75993. doi: 10.2196/75993 (PMC12519033; doi:10.2196/75993)
Supplement: Multimedia Appendix 2 [file aging_v8i1e75993_app2.docx]

| **Characteristic** | **Labels** | **Description** |
| --- | --- | --- |
| Abuse Type | ADVO breach | A person of interest violating their apprehended domestic violence order (ADVO) |
|  | Assault (unspecified) | An unspecified form of attack towards a victim reported in the DV event as bashing, clipping, assaulting, smacking. |
|  | Attempting to hit the victim with an object or weapon | Using an object to cause harm or pain to a victim (but did not occur). |
|  | Attempting to set fire to premises | An attempt to initiate a fire inside the premises (resulting in property damage). |
|  | Biting | Biting a victim. |
|  | Blocking | Physically blocking with his presence an exit or entry forcing the victim to stay in the same area |
|  | Chasing | Chasing a victim. |
|  | Choking | Attempting to strangle/or strangling a victim. |
|  | Hair pulling/dragging by the hair | Dragging a victim (either by hair or other body part). |
|  | Elbowing | Attacking a victim through their elbows. |
|  | Emotional/verbal abuse | Yelling/shouting emotional/verbal abuse towards the victim |
|  | Financial control | Controlling any financial resources that belong to the victim. |
|  | Forced entry | Attempting to physically force their way into the victim’s premises. |
|  | Gagging | Gagging a victim to avoid any cries for help. |
|  | Grabbing | Grabbing forcefully a victim from any body part or wearing accessory e.g. bag, scarf. |
|  | Hair pulling | Pulling a victim by their hair |
|  | Harassment | Harassing a victim in any way e.g. texts, emails, or appearing on the premises |
|  | Headbutting | Headbutting a victim. |
|  | Headlocking | Putting the victim in a headlock. |
|  | Hitting the victim with an object or weapon | Using an object to cause harm or pain to a victim. |
|  | Intimidation | Intimidating in any way (physically or explicitly making a threat of any kind) a victim. |
|  | Kicking | Kicking a victim. |
|  | Kneeing | Kneeing a victim. |
|  | Limb twisting | Twisting one or more body parts of a victim to cause pain or harm. |
|  | Lunging | Lunging towards a victim. |
|  | Other | Various unclassified actions by persons of interest |
|  | Ordered dog attack | Ordering a dog to attack a victim. |
|  | Physical restraining | Physically restraining a victim. |
|  | Prevent child access | Prohibiting a victim seeing their children. |
|  | Property damage | Causing damage to a victim’s property. |
|  | Possession | Wrongfully possessing items belonging to a victim e.g. cell phone, car keys. |
|  | Pulling | Physically pulling a victim from a location (but not dragging). |
|  | Punching | Punching a victim. |
|  | Pushing | Pushing a victim |
|  | Scratching | Scratching a victim |
|  | Self-harming | Using (or threatening to use) self-harm to blackmail a victim. |
|  | Sexual assault | An offender attempted to sexually assault a victim. |
|  | Shaking | Physically grabbing and shaking a victim. |
|  | Slapping | Slapping a victim. |
|  | Social restriction | Prohibiting a victim to socialise, see relatives or friends or leave the residential premises. |
|  | Spitting | Spitting at a victim. |
|  | Stabbing | Stabbing at a victim. |
|  | Stalking | Stalking a victim. |
|  | Victim being thrown around | Physically throwing the victim on the ground, floor, wall or furniture. |

*Note*. ADVO= Apprehended domestic violence order; DV = domestic violence.
