# Supplementary material for: Older Perpetrators of Domestic Violence: Mixed-Effects Logistic Regression Analysis of Police Records
Source: JMIR Aging. 2025 Sep 29;8:e75993. doi: 10.2196/75993 (PMC12519033; doi:10.2196/75993)
Supplement: Multimedia Appendix 3 [file aging_v8i1e75993_app3.docx]

| **Victim injury category** | **Specific victim injury types derived from text mining** |
| --- | --- |
| Soft tissue injury | Bruising |
|  | Lump |
|  | Red marks (on skin) |
|  | Swelling |
| Wound (non-specific) | Bleeding |
|  | Cut |
|  | Scratch |
|  | Graze |
| Soreness | Soreness |
| Fracture | Fracture |
| Burn mark | Burn mark |
| Bite mark | Bite mark |
| Stab wound | Stab wound |
| Broken tooth | Broken tooth |
| Periorbital bruising | Black eye |
| Other | Torn nail |
|  | Miscellaneous |
