# Supplementary material for: Older Perpetrators of Domestic Violence: Mixed-Effects Logistic Regression Analysis of Police Records
Source: JMIR Aging. 2025 Sep 29;8:e75993. doi: 10.2196/75993 (PMC12519033; doi:10.2196/75993)
Supplement: Multimedia Appendix 4 [file aging_v8i1e75993_app4.docx]

| Year | 55-64 Female | | | | | 55-64 Male Total | | | | |
| --- | --- | --- | --- | --- | --- | --- | --- | --- | --- | --- |
|  | Events | Persons | Physical abuse only | Non physical abuse only | Both Physical and non-physical abuse | Events | Persons | Physical abuse only | Non physical abuse only | Both Physical and non-physical abuse |
| 2005 | 62 | 49 | 23 | 6 | 12 | 508 | 370 | 220 | 37 | 80 |
| 2006 | 78 | 72 | 35 | 11 | 13 | 491 | 355 | 192 | 42 | 57 |
| 2007 | 93 | 79 | 45 | 13 | 15 | 468 | 344 | 196 | 41 | 59 |
| 2008 | 88 | 78 | 52 | 6 | 21 | 447 | 350 | 211 | 44 | 92 |
| 2009 | 86 | 74 | 43 | 4 | 13 | 488 | 363 | 230 | 33 | 97 |
| 2010 | 106 | 95 | 53 | 11 | 24 | 548 | 389 | 232 | 44 | 91 |
| 2011 | 115 | 97 | 65 | 7 | 25 | 524 | 392 | 221 | 54 | 98 |
| 2012 | 103 | 93 | 62 | 6 | 28 | 514 | 371 | 229 | 46 | 93 |
| 2013 | 123 | 109 | 59 | 11 | 25 | 568 | 400 | 257 | 50 | 113 |
| 2014 | 134 | 118 | 79 | 8 | 30 | 585 | 436 | 261 | 53 | 101 |
| 2015 | 154 | 123 | 82 | 9 | 31 | 643 | 484 | 283 | 67 | 106 |
| 2016 | 156 | 130 | 85 | 12 | 44 | 645 | 498 | 306 | 70 | 138 |
| Total | 1,298 | 1,117 | 683 | 104 | 281 | 6,429 | 4,752 | 2,838 | 581 | 1,125 |

*Note.* Abuse types are assessed at perpetrator level, not event-level and are thus unique for each participant per year
