# Supplementary material for: Older Perpetrators of Domestic Violence: Mixed-Effects Logistic Regression Analysis of Police Records
Source: JMIR Aging. 2025 Sep 29;8:e75993. doi: 10.2196/75993 (PMC12519033; doi:10.2196/75993)
Supplement: Multimedia Appendix 5 [file aging_v8i1e75993_app5.docx]

Yearly breakdown of total police-reported events by sex (female and male) for persons of interest aged 65+ years (2005-2016).

| Year | 65+ Female | | | | | 65+ Male Total | | | | |
| --- | --- | --- | --- | --- | --- | --- | --- | --- | --- | --- |
|  | Events | Persons | Physical abuse only | Non physical abuse only | Both Physical and non-physical abuse | Events | Persons | Physical abuse only | Non physical abuse only | Both Physical and non-physical abuse |
| 2005 | 20 | 19 | 12 | 2 | 0 | 138 | 119 | 68 | 11 | 25 |
| 2006 | 26 | 23 | 14 | 1 | 4 | 157 | 127 | 76 | 7 | 31 |
| 2007 | 27 | 27 | 20 | 1 | 3 | 189 | 147 | 88 | 13 | 34 |
| 2008 | 32 | 28 | 13 | 3 | 3 | 161 | 133 | 79 | 13 | 31 |
| 2009 | 38 | 30 | 18 | 1 | 7 | 182 | 152 | 92 | 17 | 34 |
| 2010 | 44 | 37 | 18 | 6 | 7 | 194 | 155 | 104 | 18 | 46 |
| 2011 | 38 | 34 | 15 | 7 | 4 | 234 | 174 | 112 | 15 | 41 |
| 2012 | 41 | 39 | 26 | 2 | 6 | 219 | 152 | 67 | 23 | 29 |
| 2013 | 51 | 40 | 23 | 7 | 7 | 259 | 192 | 121 | 26 | 52 |
| 2014 | 41 | 40 | 26 | 6 | 9 | 245 | 192 | 126 | 25 | 53 |
| 2015 | 52 | 44 | 29 | 3 | 15 | 273 | 210 | 119 | 29 | 46 |
| 2016 | 60 | 54 | 34 | 8 | 13 | 255 | 205 | 118 | 32 | 51 |
| Total | 470 | 415 | 248 | 47 | 78 | 2,506 | 1,958 | 1,170 | 229 | 473 |

*Note.* Abuse types are assessed at perpetrator level, not event level and are thus unique for each participant per year
