# Supplementary material for: Older Perpetrators of Domestic Violence: Mixed-Effects Logistic Regression Analysis of Police Records
Source: JMIR Aging. 2025 Sep 29;8:e75993. doi: 10.2196/75993 (PMC12519033; doi:10.2196/75993)
Supplement: Multimedia Appendix 6 [file aging_v8i1e75993_app6.docx]

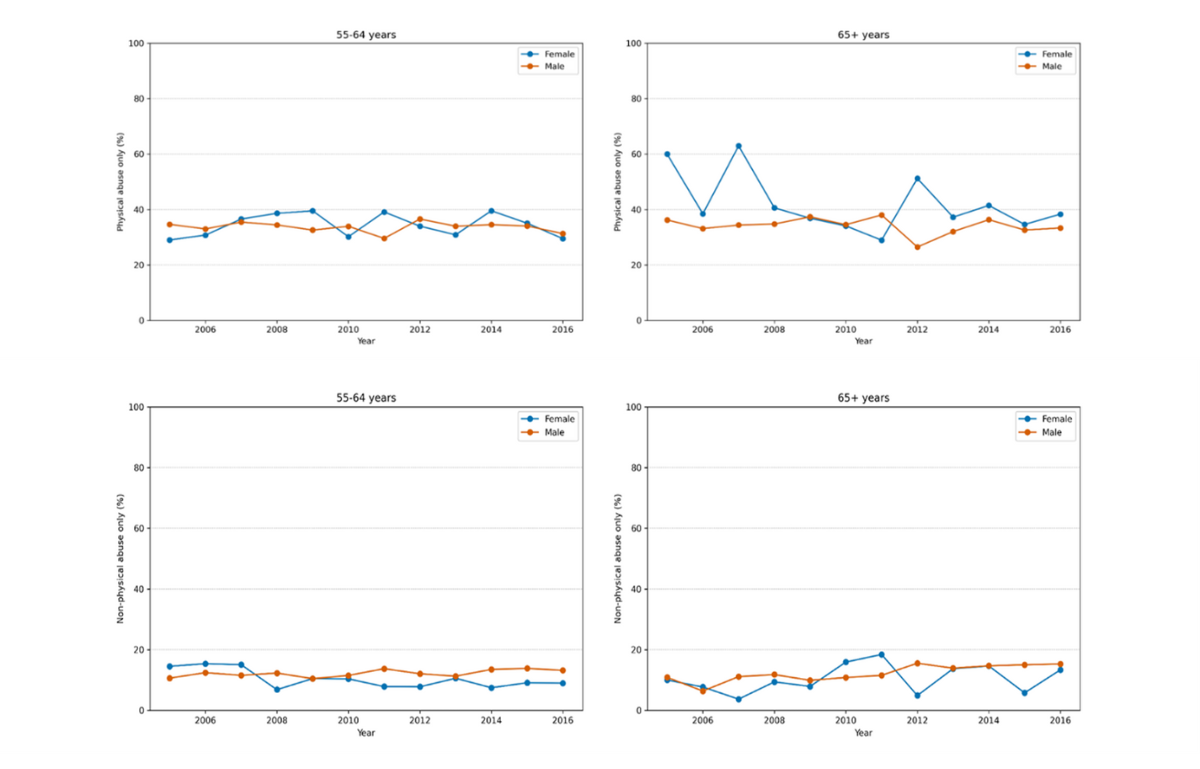


*Note.* Total events with female POIs aged 55-64: 1298; Total events with female POIs aged 65+: 470; Total events with male POIs aged 55-64: 6429; Total events with male POIs aged 65+: 2506

^a^ Person of interest, ^b^ Domestic violence
