# Supplementary figures and images for: Older Perpetrators of Domestic Violence: Mixed-Effects Logistic Regression Analysis of Police Records
Source: JMIR Aging. 2025 Sep 29;8:e75993. doi: 10.2196/75993 (PMC12519033; doi:10.2196/75993)

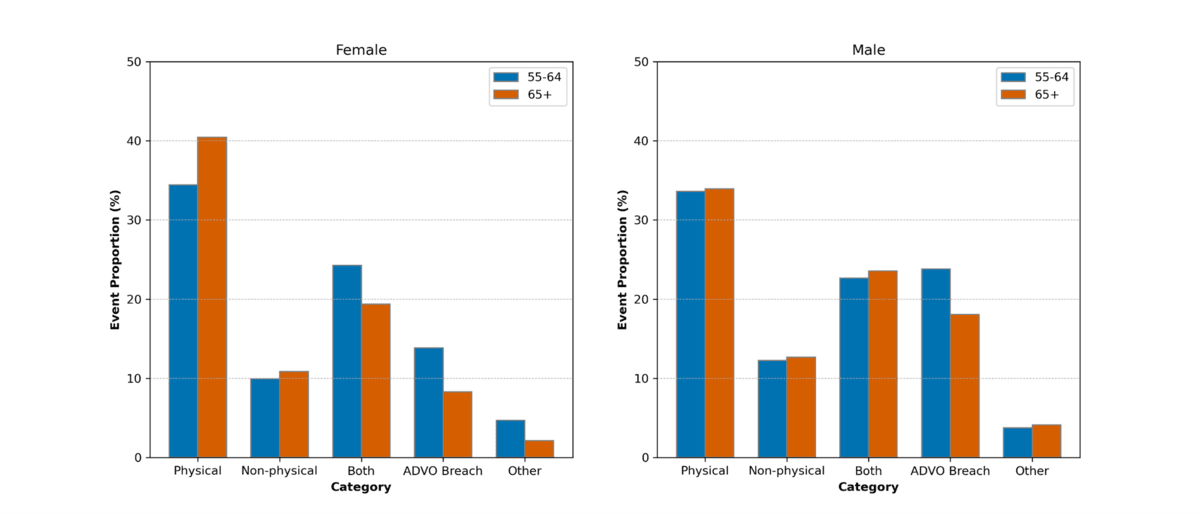

Supplement: Multimedia Appendix 7 [file aging_v8i1e75993_app7.png]
