# Supplementary material for: Older Perpetrators of Domestic Violence: Mixed-Effects Logistic Regression Analysis of Police Records
Source: JMIR Aging. 2025 Sep 29;8:e75993. doi: 10.2196/75993 (PMC12519033; doi:10.2196/75993)
Supplement: Multimedia Appendix 8 [file aging_v8i1e75993_app8.docx]

|  | POIs: 55 – 64 years | | | | POIs: 65+ years | | | |
| --- | --- | --- | --- | --- | --- | --- | --- | --- |
| Variable | aOR | Lower 95% CI | Upper 95% CI | P value | aOR | Lower 95% CI | Upper 95% CI | P value |
| Premises |  |  |  |  |  |  |  |  |
| Non-residential | 1.0 (ref) |  |  |  | 1.0 (ref) |  |  |  |
| Residential | 1.38 | 1.17 | 1.63 | 0.000 | 1.33 | 1.02 | 1.74 | 0.036 |
| Alcohol-related event |  |  |  |  |  |  |  |  |
| No | 1.0 (ref) |  |  |  | 1.0 (ref) |  |  |  |
| Yes | 1.52 | 1.34 | 1.72 | 0.000 | 1.15 | 0.93 | 1.41 | 0.189 |
| Indigenous status (victim) |  |  |  |  |  |  |  |  |
| Non-Indigenous | 1.0 (ref) |  |  |  | 1.0 (ref) |  |  |  |
| Indigenous | 1.72 | 1.18 | 2.49 | 0.004 | 0.96 | 0.42 | 2.18 | 0.922 |
| Victim injury documented |  |  |  |  |  |  |  |  |
| No | 1.0 (ref) |  |  |  | 1.0 (ref) |  |  |  |
| Yes | 3.28 | 2.88 | 3.75 | 0.000 | 2.86 | 2.35 | 3.48 | 0.000 |
| Indigenous status (POI) |  |  |  |  |  |  |  |  |
| Non-Indigenous | 1.0 (ref) |  |  |  | 1.0 (ref) |  |  |  |
| Indigenous | 0.68 | 0.49 | 0.95 | 0.022 | 1.60 | 0.71 | 3.58 | 0.258 |
| POI sex |  |  |  |  |  |  |  |  |
| Male | 1.0 (ref) |  |  |  | 1.0 (ref) |  |  |  |
| Female | 1.22 | 1.04 | 1.44 | 0.016 | 1.22 | 0.95 | 1.57 | 0.126 |

*Note*: aOR = Adjusted Odds Ratio; CI = 95% Confidence Interval. All ORs and CIs are rounded to two decimal places, and P values to three decimal places.

Model for aOR estimates adjusted for all variables presented in Table 3. Only factors significant in at least one age group are presented.
