# Supplementary material for: Older Perpetrators of Domestic Violence: Mixed-Effects Logistic Regression Analysis of Police Records
Source: JMIR Aging. 2025 Sep 29;8:e75993. doi: 10.2196/75993 (PMC12519033; doi:10.2196/75993)
Supplement: Multimedia Appendix 9 [file aging_v8i1e75993_app9.docx]

|  | POIs: 55 – 64 years | | | | POIs: 65+ years | | | |
| --- | --- | --- | --- | --- | --- | --- | --- | --- |
| Variable | aOR | Lower 95% CI | Upper 95% CI | P value | aOR | Lower 95% CI | Upper 95% CI | P value |
| Premises |  |  |  |  |  |  |  |  |
| Non-residential | 1.0 (ref) |  |  |  | 1.0 (ref) |  |  |  |
| Residential | 1.35 | 1.12 | 1.63 | 0.002 | 1.72 | 1.21 | 2.45 | 0.002 |
| Alcohol-related event |  |  |  |  |  |  |  |  |
| No | 1.0 (ref) |  |  |  | 1.0 (ref) |  |  |  |
| Yes | 1.25 | 1.11 | 1.41 | 0.000 | 1.12 | 0.89 | 1.40 | 0.338 |
| Victim sex |  |  |  |  |  |  |  |  |
| Male | 1.0 (ref) |  |  |  | 1.0 (ref) |  |  |  |
| Female | 1.20 | 1.03 | 1.39 | 0.016 | 1.26 | 0.98 | 1.64 | 0.076 |
| Victim injury documented |  |  |  |  |  |  |  |  |
| No | 1.0 (ref) |  |  |  | 1.0 (ref) |  |  |  |
| Yes | 1.63 | 1.45 | 1.84 | 0.000 | 1.56 | 1.28 | 1.91 | 0.000 |
| Indigenous status (POI) |  |  |  |  |  |  |  |  |
| Non-Indigenous | 1.0 (ref) |  |  |  | 1.0 (ref) |  |  |  |
| Indigenous | 0.62 | 0.44 | 0.88 | 0.008 | 1.34 | 0.58 | 3.08 | 0.498 |
| POI sex |  |  |  |  |  |  |  |  |
| Male | 1.0 (ref) |  |  |  | 1.0 (ref) |  |  |  |
| Female | 1.21 | 1.03 | 1.43 | 0.019 | 0.81 | 0.60 | 1.10 | 0.175 |
| Substance use disorder (POI) |  |  |  |  |  |  |  |  |
| Not present | 1.0 (ref) |  |  |  | 1.0 (ref) |  |  |  |
| Present | 1.87 | 1.36 | 2.56 | 0.000 | 1.98 | 1.13 | 3.45 | 0.017 |
| Dementia (POI) |  |  |  |  |  |  |  |  |
| Not present | 1.0 (ref) |  |  |  | 1.0 (ref) |  |  |  |
| Present | 0.81 | 0.31 | 2.14 | 0.672 | 1.73 | 1.19 | 2.53 | 0.004 |
| Childhood-onset disorder (POI) |  |  |  |  |  |  |  |  |
| Not present | 1.0 (ref) |  |  |  | 1.0 (ref) |  |  |  |
| Present | 1.19 | 0.69 | 2.04 | 0.532 | 2.52 | 1.05 | 6.05 | 0.039 |

aOR = Adjusted Odds Ratio; CI = 95% Confidence Interval. All ORs and CIs are rounded to two decimal places, and P values to three decimal places.

Model for aOR estimates adjusted for all variables presented in Table 3. Only factors significant in at least one age group are presented.
