# Supplementary material for: Older Perpetrators of Domestic Violence: Mixed-Effects Logistic Regression Analysis of Police Records
Source: JMIR Aging. 2025 Sep 29;8:e75993. doi: 10.2196/75993 (PMC12519033; doi:10.2196/75993)
Supplement: Multimedia Appendix 10 [file aging_v8i1e75993_app10.docx]

|  | | | | | | | | |
| --- | --- | --- | --- | --- | --- | --- | --- | --- |
|  | POIs: 55 – 64 years | | | | POIs: 65+ years | | | |
| Variable | aOR | Lower 95% CI | Upper 95% CI | P value | aOR | Lower 95% CI | Upper 95% CI | P value |
| Alcohol-related event |  |  |  |  |  |  |  |  |
| No | 1.0 (ref) |  |  |  | 1.0 (ref) |  |  |  |
| Yes | 0.91 | 0.78 | 1.08 | 0.293 | 1.68 | 1.28 | 2.20 | 0.000 |
| Victim age |  |  |  |  |  |  |  |  |
|  | 1.01 | 1.00 | 1.02 | 0.006 | 1.01 | 0.99 | 1.01 | 0.206 |
| Victim injury documented |  |  |  |  |  |  |  |  |
| No | 1.0 (ref) |  |  |  | 1.0 (ref) |  |  |  |
| Yes | 0.44 | 0.37 | 0.53 | 0.000 | 0.47 | 0.35 | 0.62 | 0.000 |
| Substance use disorder (victim) |  |  |  |  |  |  |  |  |
| Not present | 1.0 (ref) |  |  |  | 1.0 (ref) |  |  |  |
| Present | 2.15 | 1.03 | 4.48 | 0.041 | 0.82 | 0.16 | 4.24 | 0.815 |
| Indigenous status (POI) |  |  |  |  |  |  |  |  |
| Non-Indigenous | 1.0 (ref) |  |  |  | 1.0 (ref) |  |  |  |
| Indigenous | 1.52 | 1.02 | 2.27 | 0.042 | 1.40 | 0.48 | 4.12 | 0.537 |
| POI sex |  |  |  |  |  |  |  |  |
| Male | 1.0 (ref) |  |  |  | 1.0 (ref) |  |  |  |
| Female | 0.72 | 0.57 | 0.90 | 0.005 | 0.98 | 0.68 | 1.41 | 0.912 |

*Note*. aOR = Adjusted Odds Ratio; CI = 95% Confidence Interval. All ORs and CIs are rounded to two decimal places, and P values to three decimal places.

Model for aOR estimates adjusted for all variables presented in Table 3. Only factors significant in at least one age group are presented.
